# HLA-Bw4-I-80 Isoform Differentially Influences Clinical Outcome As Compared to HLA-Bw4-T-80 and HLA-A-Bw4 Isoforms in Rituximab or Dinutuximab-Based Cancer Immunotherapy

**DOI:** 10.3389/fimmu.2017.00675

**Published:** 2017-06-12

**Authors:** Amy K. Erbe, Wei Wang, Patrick K. Reville, Lakeesha Carmichael, KyungMann Kim, Eneida A. Mendonca, Yiqiang Song, Jacquelyn A. Hank, Wendy B. London, Arlene Naranjo, Fangxin Hong, Michael D. Hogarty, John M. Maris, Julie R. Park, M. F. Ozkaynak, Jeffrey S. Miller, Andrew L. Gilman, Brad Kahl, Alice L. Yu, Paul M. Sondel

**Affiliations:** ^1^Department of Human Oncology, University of Wisconsin, Madison, WI, United States; ^2^Department of Biostatistics and Medical Informatics, University of Wisconsin, Madison, WI, United States; ^3^Dana-Farber Cancer Institute/Boston Children’s Cancer and Blood Disorder Center, Harvard Medical School, Boston, MA, United States; ^4^COG Statistics and Data Center, Department of Biostatistics, University of Florida, Gainesville, FL, United States; ^5^Department of Biostatistics, Harvard University, Dana Farber Cancer Institute, Boston, MA, United States; ^6^Children’s Hospital of Philadelphia, University of Pennsylvania School of Medicine, Philadelphia, PA, United States; ^7^Provenance Biopharmaceuticals, Carlisle, MA, United States; ^8^Seattle Children’s Hospital/University, Seattle, WA, United States; ^9^University of Washington, Seattle, WA, United States; ^10^New York Medical College, Valhalla, NY, United States; ^11^Department of Medicine, University of Minnesota, Minneapolis, MN, United States; ^12^Levine Children’s Hospital, Charlotte, NC, United States; ^13^Department of Medicine, Washington University, St. Louis, MO, United States; ^14^Department of Pediatrics, Hematology/Oncology, Moores Cancer Center, University of California San Diego, San Diego, CA, United States; ^15^Institute of Stem Cell and Translational Cancer Research, Chang Gung Memorial Hospital, Taoyuan, Taiwan; ^16^Department of Pediatrics, University of Wisconsin-Madison, Madison, WI, United States

**Keywords:** KIR, KIR-ligand, HLA-Bw4, HLA, MHC class I, natural killer cells, cancer immunotherapy

## Abstract

Killer-cell immunoglobulin-like receptors (KIRs) are a family of glycoproteins expressed primarily on natural killer cells that can regulate their function. Inhibitory KIRs recognize MHC class I molecules (KIR-ligands) as ligands. We have reported associations of KIRs and KIR-ligands for patients in two monoclonal antibody (mAb)-based trials: (1) A Children’s Oncology Group (COG) trial for children with high-risk neuroblastoma randomized to immunotherapy treatment with dinutuximab (anti-GD2 mAb) + GM-CSF + IL-2 + isotretinion or to treatment with isotretinoin alone and (2) An Eastern Cooperative Oncology Group (ECOG) trial for adults with low-tumor burden follicular lymphoma responding to an induction course of rituximab (anti-CD20 mAb) and randomized to treatment with maintenance rituximab or no-maintenance rituximab. In each trial, certain KIR/KIR-ligand genotypes were associated with clinical benefit for patients randomized to immunotherapy treatment (immunotherapy in COG; maintenance rituximab in ECOG) as compared to patients that did not receive the immunotherapy [isotretinoin alone (COG); no-maintenance (ECOG)]. Namely, patients with both KIR3DL1 and its HLA-Bw4 ligand (KIR3DL1+/HLA-Bw4+ genotype) had improved clinical outcomes if randomized to immunotherapy regimens, as compared to patients with the KIR3DL1+/HLA-Bw4+ genotype randomized to the non-immunotherapy regimen. Conversely, patients that did not have the KIR3DL1+/HLA-Bw4+ genotype showed no evidence of a difference in outcome if receiving the immunotherapy vs. no-immunotherapy. For each trial, HLA-Bw4 status was determined by assessing the genotypes of three separate isoforms of HLA-Bw4: (1) HLA-B-Bw4 with threonine at amino acid 80 (B-Bw4-T80); (2) HLA-B-Bw4 with isoleucine at amino acid 80 (HLA-B-Bw4-I80); and (3) HLA-A with a Bw4 epitope (HLA-A-Bw4). Here, we report on associations with clinical outcome for patients with KIR3DL1 and these separate isoforms of HLA-Bw4. Patients randomized to immunotherapy with KIR3DL1+/A-Bw4+ or with KIR3DL1+/B-Bw4-T80+ had better outcome vs. those randomized to no-immunotherapy, whereas for those with KIR3DL1+/B-Bw4-I80+ there was no evidence of a difference based on immunotherapy vs. no-immunotherapy. Additionally, we observed differences within treatment types (either within immunotherapy or no-immunotherapy) that were associated with the genotype status for the different KIR3DL1/HLA-Bw4-isoforms. These studies suggest that specific HLA-Bw4 isoforms may differentially influence response to these mAb-based immunotherapy, further confirming the involvement of KIR-bearing cells in tumor-reactive mAb-based cancer immunotherapy.

## Introduction

One modality of cancer immunotherapy utilizes tumor-reactive monoclonal antibodies (mAbs) to elicit a tumor-targeted immune response. Two recently completed clinical trials, in separate disease settings, utilized tumor-reactive mAbs to successfully target and treat the tumors: (1) the combination of dinutuximab with IL-2, GM-CSF, and isotretinoin for patients with high-risk neuroblastoma (1) and (2) rituximab for the treatment of patients with low-tumor burden follicular lymphoma (FL) (2).

Natural killer (NK) cells can contribute to the response to tumor-reactive mAb-based immunotherapeutics through antibody-dependent cellular cytotoxicity (ADCC). The ability of NK cells to elicit ADCC is regulated by activating and inhibiting signaling. Killer-cell immunoglobulin-like receptors (KIRs) are a class of receptors expressed on NK cells that influence such signaling (3, 4). Most inhibitory KIRs interact with HLA class I molecules as their ligands (KIR-ligand) (5). Specifically, KIR2DL1 binds to HLA-C2, KIR2DL2 and KIR2DL3 bind to HLA-C1, and KIR3DL1 recognizes the Bw4 epitope of HLA-A and HLA-B (6, 7). The independent segregation and inheritance of KIRs and KIR-ligands help to shape NK cell function and response to immunotherapeutic agents (8–11). When inhibitory KIRs interact with class I HLA molecules on target cells, NK cell-mediated lysis and ADCC are inhibited. During development, KIR/KIR-ligand interactions lead to self tolerance and NK cells become “licensed NK cells” (12–14). Licensed NK cells have augmented cytotoxicity against class I negative tumors compared to unlicensed NK cells (15, 16).

Killer-cell immunoglobulin-like receptors and KIR-ligands segregate independently: KIR genes are located on chromosome 19; HLA genes (KIR-ligands) are located on chromosome 6. Several studies have shown that genotypic differences of KIR and KIR-ligands can influence clinical outcome of certain cancer immunotherapies (8, 11, 17–19). We recently showed in two clinical trials that KIR3DL1 and its KIR-ligand, HLA-Bw4, appear to influence clinical outcome.

In a phase III trial (ANBL0032) of high-risk neuroblastoma patients, conducted by the Children’s Oncology Group (COG) (1), patients who inherited the KIR3DL1 gene and the gene for its HLA-Bw4 ligand (KIR3DL1+/Bw4+ genotype) and were treated with an immunotherapy regimen [dinutuximab (anti-GD2), IL-2, GM-CSF, and isotretinoin] had improved event-free survival (EFS) and overall survival as compared to those treated with isotretinoin alone (20, 21). In a separate Eastern Cooperative Oncology Group (ECOG) Phase III clinical trial of low-tumor burden FL (2), patients who were KIR3DL1+/HLA-Bw4+ and treated with a continuous regimen of maintenance rituximab had improved duration of response and % tumor shrinkage compared to KIR3DL1+/HLA-Bw4+ patients who were randomized to not receive maintenance rituximab (22, 23). Conversely, we did not observe improved outcome for patients that were *not* KIR3DL1+/HLA-Bw4+ when randomized to immunotherapy, in either study (22, 23). Furthermore, in both the COG and ECOG studies, patients who were randomized to the immunotherapy regimen that were KIR3DL1+/HLA-Bw4+ had better outcome compared to patients who were *not* KIR3DL1+/HLA-Bw4+.

Given these similar associations with outcome for the KIR3DL1/HLA-Bw4 interaction in these two clinical trials, we chose to evaluate these more deeply by evaluating the potential influence of distinct HLA-Bw4 isoforms. Polymorphisms in the α1 helix (positions 77–83) of HLA class I correspond to the sequence site of the Bw4 epitope that is recognized by KIR3DL1 (24). In KIR/KIR-ligand associations, we analyzed in these COG and ECOG trials, individuals were considered positive for HLA-Bw4 if they were found to have at least one of the three isoforms of HLA-Bw4: (1) HLA-B allele with a threonine at amino acid position 80 (B-Bw4-T80), (2) HLA-B allele with an isoleucine at amino acid position 80 (B-Bw4-I80), or (3) HLA-A with a Bw4 epitope (A-Bw4). Patients were negative for HLA-Bw4 if they did not have any of these three isoforms. These polymorphisms of this Bw4 epitope can impact KIR3DL1 recognition (25–29). As such, we describe the impact of the genotype status of B-Bw4-T80, B-Bw4-I80, and A-Bw4, together with the genotype status of KIR3DL1, on the clinical outcome, based on a clinical outcome parameter that measured the duration of response to the treatment regimen (EFS in COG; duration of response in ECOG).

## Materials and Methods

### Patients

#### COG ANBL0032 Patients

The phase III neuroblastoma clinical trial (ANBL0032; Clinicaltrials.gov # NCT00026312) evaluated the efficacy of isotretinoin alone as compared to an immunotherapeutic regimen consisting of dinutuximab (anti-GD2), aldesleukin (IL-2), sargramostim (GM-CSF), and isotretinoin (1). Of the 226 patients randomized, 174 patients (immunotherapy: *n* = 88; isotretinoin: *n* = 86) had DNA available, allowing evaluation of KIR/KIR-ligand genotype association with updated clinical outcome (>5-year follow-up if no event). All analyses in this study were conducted utilizing an intent-to-treat approach. All patients signed IRB approved consent forms enabling lab-based immune correlative analyses, and the genotyping done at UW-Madison was approved by the UW-IRB.

#### ECOG E4402 Patients

The Phase III ECOG clinical trial (E4402; ClinicalTrials.gov #NCT00075946) evaluated the efficacy of single agent, rituximab therapy for adults with low-tumor burden FL. Clinical results from this study have been reported elsewhere (2). A total of 408 patients with FL were entered, with 289 patients responding and randomized to no-maintenance or maintenance rituximab regimens. Disease measurements were obtained every 13 weeks (2). Of the 289 randomized patients from this trial, 213 patients had evaluable DNA and clinical data for this study, and 159 of them were randomized to no-maintenance (*n* = 80) or maintenance rituximab (*n* = 79) treatment. Of these 79 patients treated with maintenance rituximab, 75 patients had clinical data available for duration of response. All patients signed IRB approved consent forms enabling lab-based immune correlative analyses, and the genotyping done at UW-Madison was approved by the UW-IRB.

### Genotyping

KIR3DL1 gene status was determined by a SYBR green real-time PCR reaction (30, 31). The genotype for HLA-Bw4, which includes three known HLA-Bw4 epitopes (B-Bw4-T80, B-Bw4-I80, and A-Bw4) were determined by PCR-SSP reactions using the KIR HLA Ligand SSP typing kit (product number 104.201-12u from Olerup, West Chester, PA, USA) with GoTaq DNA polymerase (M8295, Promega, WI, USA). All genotyping was conducted in a blinded manner, whereby individuals who determined the genotype of the patients did not have access to the clinical outcome data.

### Statistical Methods

The goal of these analyses was to evaluate the association of KIR3DL1 in combination with each HLA-Bw4 isoform (B-Bw4-T80, B-Bw4-I80, and A-Bw4) on response to therapy (EFS or duration of response). For the COG trial, EFS time was defined as the time from study enrollment until the first occurrence of relapse, progressive disease, secondary cancer, or death or until the last contact with the patient if none of these events occurred (censored). For the ECOG trial, the duration of response was defined as the time from randomization (following an initial response to the induction rituximab treatment) to documented disease progression (2).

Cox proportional hazards regression models and log-rank tests were used to compare EFS/duration of response curves by treatment and genotype combinations. The proportional hazards assumption was tested, and when the assumption was not met, adjustments were made by incorporating time-dependent covariates into the model. For both trials, only randomized patients were included in the analyses. Statistical analyses were performed using SAS v9.4 (SAS Institute, Cary, NC, USA).

## Results

### HLA-Bw4 Isoforms, Together with KIR3DL1, Differentially Influence the Impact of mAb-Based Immunotherapy on Clinical Outcome of Neuroblastoma Patients

In our analyses of associations of KIR/KIR-ligand genotypic influence on clinical response in the neuroblastoma study (ANBL0032), we reported on differences in clinical outcome for those KIR3DL1+/Bw4+ (immunotherapy *n* = 58; isotretinoin *n* = 61) and those *not* KIR3DL1+/Bw4+ (immunotherapy *n* = 30; isotretinoin *n* = 25), and differences in response were observed dependent upon treatment type (20, 21). Since not all of the isoforms of HLA-Bw4 may interact with KIR3DL1 to the same degree, we further assessed patients with different HLA-Bw4 isoforms in this setting.

**Figure 1:**
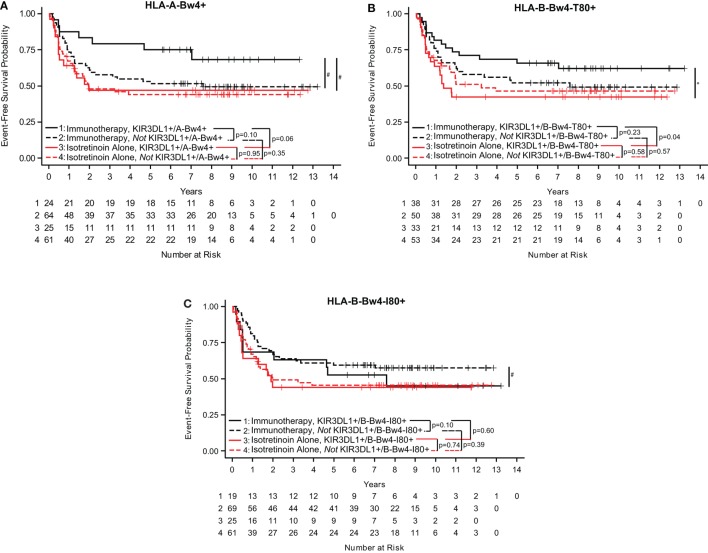
KIR3DL1/HLA-Bw4 isoforms differentially influence event-free survival (EFS) in neuroblastoma patients. In neuroblastoma patients treated with immunotherapy (black lines) or isotretinoin alone (red lines), genotype statuses of KIR3DL1/A-Bw4 **(A)**, KIR3DL1/B-Bw4-T80 **(B)**, and KIR3DL1/B-Bw4-I80 **(C)** had differential effects on EFS. Those with the genes present are represented by solid lines, and those without the genes present are represented by dotted lines. The number of patients at risk for EFS at a given time point are provided below the *x*-axis. *p < 0.5 and #*p* < 0.10.

To better understand the KIR/KIR-ligand genotypic influence on clinical outcome, we evaluated the effect of Bw4 epitope on either an HLA-A or HLA-B allele. In this study, patients who were KIR3DL1+/A-Bw4+ had a trend toward improved EFS if they were treated with immunotherapy as compared to those treated with isotretinoin alone (*p* = 0.06; Figure 1A) (Table S1 in Supplementary Material). In contrast, we did not find a significant difference in EFS for patients receiving the immunotherapy vs. those randomized to not receive the immunotherapy (i.e., isotretinoin alone) in the patients that were *not* KIR3DL1+/A-Bw4+ (*p* = 0.35; Figure 1A).

We found that B-Bw4-T80 and B-Bw4-I80 differentially influenced EFS in these neuroblastoma patients (Table S1 in Supplementary Material). Similar to results in Figure 1A, patients who were KIR3DL1+/B-Bw4-T80+ showed significantly improved EFS if they received immunotherapy compared with isotretinoin alone (*p* = 0.04; Figure 1B), whereas those that were *not* KIR3DL1+/B-Bw4-T80+ showed no difference in EFS for patients receiving the immunotherapy vs. those randomized to receive isotretinoin alone (*p* = 0.57; Figure 1B). However, for B-Bw4-I80+, the results were converse. Patients who were KIR3DL1+/B-Bw4-I80+ showed no sign of improved EFS if they received immunotherapy compared with isotretinoin alone (*p* = 0.60; Figure 1C). Furthermore, and in contrast to results in Figures 1A,B, while not significant, there appears to be improved EFS for patients receiving the immunotherapy vs. isotretinoin alone in the patients who were *not* KIR3DL1+/B-Bw4-I80+ (*p* = 0.10; Figure 1C).

These findings suggest that the different isoforms of HLA-Bw4 differentially influence the impact of anti-GD2-based immunotherapy on EFS for high-risk neuroblastoma patients.

### HLA-Bw4 Isoforms, Together with KIR3DL1, Differentially Influence the Impact of mAb-Based Immunotherapy on Clinical Outcome of FL Patients

The ECOG E4402 Phase III clinical trial sought to optimize the rituximab treatment regimen for low-tumor burden FL patients (2). As such, different from the design of the neuroblastoma COG trial described above where one treatment arm was treated with immunotherapy and the other was not, in E4402 *all* patients were initially treated with rituximab. In E4402, all FL patients received induction rituximab, consisting of four weekly rituximab treatments. After 13 weeks, those patients who achieved ≥50% tumor shrinkage were randomized to two separate treatment regimens: (1) “maintenance” rituximab was given every 13 weeks or (2) “no-maintenance” where rituximab was given only upon disease progression (2). Thus, for the parameter of disease progression, the no-maintenance group received no rituximab between randomization and disease progression. Similar to the COG findings regarding the genotype status of KIR3DL1/Bw4, in this ECOG study, we also found that those KIR3DL1+/Bw4+ (maintenance *n* = 49; no-maintenance *n* = 53) had different clinical outcome than those not KIR3DL1+/Bw4+ (maintenance *n* = 27; no-maintenance *n* = 26), which was also influenced by the treatment arm.

**Figure 2:**
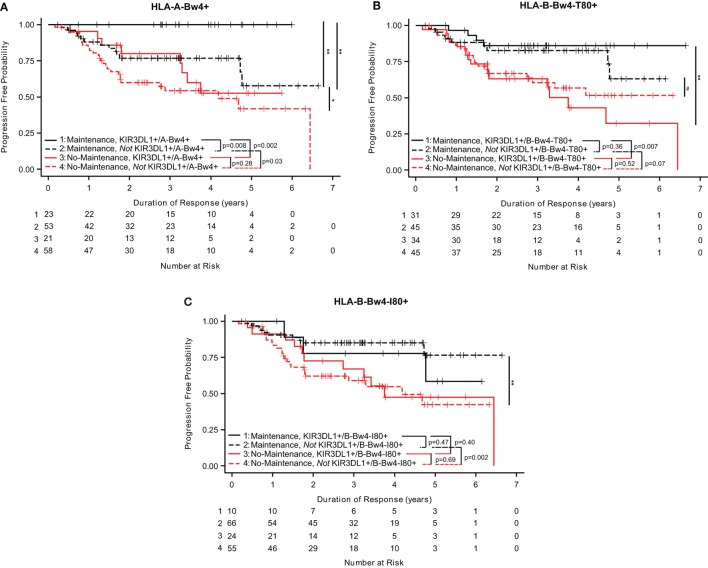
KIR3DL1/HLA-Bw4 isoforms differentially influence duration of response in follicular lymphoma (FL) patients. In FL patients treated with maintenance rituximab (red lines) or no-maintenance rituximab (black lines), genotype statuses of KIR3DL1/A-Bw4 **(A)**, KIR3DL1/B-Bw4-T80 **(B)**, and KIR3DL1/B-Bw4-I80 **(C)** had differential effects on duration of response. Those with the genes present are represented by solid lines, and those without the genes present are represented by dotted lines. The number of patients at risk for duration of response at a given time point are provided below the *x*-axis. **p < 0.01, *p < 0.5, and #*p* < 0.10.

Analyses of the three separate HLA-Bw4 isoforms suggest that the isoforms of HLA-Bw4 differently influenced the impact of maintenance rituximab. FL patients who were KIR3DL1+/A-Bw4+ that were treated with maintenance rituximab had a longer duration of response (0 of 23 progressed, Figure 2A) as compared to patients who were *not* KIR3DL1+/A-Bw4+ [13 out of 53 progressed (*p* = 0.008, Figure 2A) (Table S1 in Supplementary Material)]. Separately, patients who were KIR3DL1+/B-Bw4-T80+ also showed significantly prolonged duration of response if they received maintenance as compared with no-maintenance rituximab (*p* = 0.007; Figure 2B). In addition, those patients whowere *not* KIR3DL1+/B-Bw4-T80+ had a trend toward improved duration of response if treated with maintenance as compared with no-maintenance rituximab (*p* = 0.07; Figure 2B) (Table S1 in Supplementary Material). However, patients who were KIR3DL1+/B-Bw4-I80+ did not show prolonged duration of response if they received maintenance as compared with no-maintenance rituximab (*p* = 0.40; Figure 2C). Similar to the trends for improved EFS observed in neuroblastoma patients treated with immunotherapy (Figure 1C), those FL patients who were *not* KIR3DL1+/B-Bw4-I80+ had improved duration of response if treated with maintenance rituximab as compared to no-maintenance (*p* = 0.002; Figure 2C) (Table S1 in Supplementary Material).

These findings suggest that the different isoforms of HLA-Bw4 differentially influence the impact of rituximab maintenance treatment for these low-tumor burden FL.

## Discussion

In both of these clinical trials, in separate disease settings, tumor-reactive mAbs were used to treat the tumors. In the analysis of KIR/KIR-ligand genotypes in each of these studies, we found similar associations with outcomes based upon the influence of KIR3DL1/HLA-Bw4. Specifically, those patients who had both KIR3DL1 and HLA-Bw4 had improved clinical outcomes if they were treated with either the COG immunotherapy regimen or the maintenance rituximab regimen in ECOG as compared to those who did not receive these same immunotherapeutic regimens (20, 21). Here, we report on the analyses of the specific HLA-Bw4 isoforms in both trials. In the ECOG trial of FL patients, patients with a KIR3DL1+/A-Bw4+ genotype or a KIR3DL1+/B-Bw4-T80+ genotype showed improved outcome when randomized to the maintenance regimen rather than to the no-maintenance regimen. In contrast, patients with a KIR3DL1+/B-Bw4-I80+ genotype showed no evidence of improved outcome when randomized to the maintenance treatment vs. no-maintenance regimen. We also observed similar trends for these same analyses in the COG trial of neuroblastoma patients.

Although other mechanisms, such as antibody-dependent cellular phagocytosis and complement-dependent cellular cytotoxicity (32, 33), could also contribute to the anti-tumor efficacy of tumor antigen-specific monoclonal antibodies, we hypothesize that the anti-tumor effect of rituximab and dinutuximab in these FL and neuroblastoma patients, respectively, is primarily through ADCC. NK cells are major contributors to ADCC, and their activity is regulated *via* the interactions between KIRs/KIR-ligands (34). As such, we hypothesize that the KIR/KIR-ligand genotypes could influence the degree that patients respond to antibody-based immunotherapies. Besides NK cells, KIRs are also expressed by a subset of T cells as well as NKT cells (35, 36). Therefore, it is possible that these other cell types may also be influenced by KIR/KIR-ligand genotypes.

Besides inherited genetic differences in KIR and KIR-ligand genotypes, other individual genetic differences, such as polymorphisms in Fc gamma receptors (FCGRs), may influence patient outcome to immunotherapy. FCGR polymorphisms can alter the affinity of FCGRs for the Fc portion of antibodies (mAbs or endogenous antibodies) (37). For example, in a separate study of patients with metastatic renal cell carcinoma treated with high-dose IL-2, we found that patients with a “higher affinity” FCGR genotype had improved clinical outcome as compared to those patients with a “lower affinity” FCGR genotype (38). In our analysis of those same metastatic renal cell carcinoma patients for KIR/KIR-ligand genotype influence on outcome, we did not observe differences in clinical outcome associated with KIR3DL1 and HLA-Bw4 genotype status (39). The influence of FCGR polymorphisms on clinical outcome to rituximab is variable (40–42). For the FL patients analyzed here from this ECOG study, Kenkre and colleagues reported no association of FCGR genotype polymorphisms with patient outcome (43). In addition, some groups have found associations of FCGR genotype with clinical outcome for patients treated with anti-GD2 immunotherapy (8, 44, 45). For the neuroblastoma patients from this COG trial, FCGR genotype associations with clinical outcome are still under investigation. In addition, it has been reported that the influence from KIR/KIR-ligand interactions on NK cells may be affected by the affinity of the Fc portion of different therapeutic mAb used (46), the rituximab used in this ECOG trial and the dinutuximab used in this COG trial have similar human IgG1 Fc components, which may also help account for why we observed similar influences from HLA-Bw4 epitopes in these two separate studies where two different therapeutic mAbs were used.

These clinical data are consistent with the B-Bw4-I80 isoform functioning somewhat differently than the B-Bw4-T80 or A-Bw4 isoforms, and potentially making the tumor cells less responsive to the potential benefit of the anti-GD2 or anti-CD20 mAb-based immunotherapy. *In vitro* analyses have shown that a subset of HLA-Bw4 alleles (those with an B-Bw4-I80 isoform) show relative protection from lysis by NK cells (47, 48). The data presented here are consistent with these *in vitro* results; mAb-based immunotherapy may provide more benefit for patients with weaker NK cell inhibition from B-Bw4-T80 or A-Bw4, than for patients with stronger NK inhibition from B-Bw4-I80.

Given that patients assessed in either trial could be positive for more than one of the HLA-Bw4 epitopes, we did consider whether the HLA-Bw4 epitopes were in linkage disequilibrium. We found that A-Bw4 was not in linkage disequilibrium with either B-Bw4-I80 or B-Bw4-T80 (Table S2 in Supplementary Material). Thus, the influence that each of these HLA-Bw4 epitopes had on the length of patient response in either trial is presumably not due to linkage disequilibrium with each other.

We also considered whether the interaction of KIR3DL1 with these three different HLA-Bw4 isoforms showed any association of outcome among patients randomized to receive the immunotherapy regimens. Within the COG study, we observed a trend for improved outcome for those KIR3DL1+/HLA-A-Bw4+ vs. those not KIR3DL1+/HLA-A-Bw4+ (Figure 1A), and we also observed a trend in the opposite direction for HLA-Bw4-I80, namely, there was a trend for improved outcome for those not KIR3DL1+/HLA-B-Bw4-I80+ vs. those who were KIR3DL1+/HLA-B-Bw4-I80+ (Figure 1C). Although only a trend, this difference in Figure 1A and Figure 1C is consistent with differential function of HLA-A-Bw4 and HLA-B-Bw4-I80. No significant differences or trends were noted when we evaluated among the FL patients randomized to receive the maintenance rituximab regimen (Figures 2A–C).

The interaction of KIR3DL1 with the Bw4 epitope is dependent not only on the architecture of Bw4 but also on the sequence of the bound peptide (25, 28, 49–51). Additionally, the differences we observed between A-Bw4, B-Bw4-T80, and B-Bw4-I80 may be due to the different inhibition strength for KIR3DL1 from these isoforms. For instance, HLA-A*32:01, HLA-B*51:01, and HLA-B*58:01 strongly inhibit target cells from lysis by KIR3DL1+ NK cells, yet HLA-B*15:13 and HLA-B*27:05 have weaker inhibitory effects, despite all being HLA-Bw4 alleles (6, 25, 26, 48, 52–54). In addition, depending on the KIR3DL1 allele, expression of KIR3DL1 can vary; different HLA-A-Bw4 alleles have differential affinity for KIR3DL1 that is attributed to high vs. low expression of KIR3DL1 (55). Furthermore, the specific Bw4 allele, as well as the KIR3DL1 allele, the strength of KIR3DL1/HLA-Bw4 interaction and the binding avidity can vary (29). For example, Saunders et al. recently showed that HLA-A*24:02 acts as a poor ligand for KIR3DL1, and the strength of its interaction with KIR3DL1 differed depending on the allele of KIR3DL1 (29). The genotyping methodology employed for analyzing the many patients in these two clinical trials reported here was not able to address these more subtle allele-specific or peptide-related issues.

Another possible cause of the differences observed in these HLA-Bw4 isoforms may be due to genetic polymorphisms of KIR3DL1 (26, 28, 29, 56–60). More than 100 alleles of KIR3DL1 have been described. Phylogenetically, these alleles span three lineages based on the polymorphism of the three extracellular domains (D0–D1–D2) (53, 61). In both of these clinical studies analyzed, we did not determine the allelic differences of the KIR genes, but rather we determined their presence or absence. Thus, we cannot assess how different KIR3DL1 alleles may affect the interactions between different isoforms of HLA-Bw4. We did, however, assess if KIR3DL1 allelic status could influence the interactions of KIR3DL1 with HLA-Bw4 and with the separate HLA-Bw4 isoforms. KIR3DL1 and KIR3DS1 are alleles, thus individuals can have 2, 1, or 0 copies of KIR3DL1 (2 copies: KIR3DL1/KIR3DL1, 1 copy: KIR3DL1/KIR3DS1, or 0 copies: KIR3DS1/KIRDS1). Although KIR3DS1 has not been shown to utilize HLA-Bw4 as a ligand *in vitro*, whether KIR3DS1 may still interact with HLA-Bw4 *in vivo* is controversial (62–65). We assessed whether the allelic status of KIR3DL1/KIR3DS1 together with HLA-Bw4 (and HLA-Bw4 isoforms) influenced patient response. We found that there was no evidence of an association with outcome in either the COG or the ECOG study that could be linked to the allelic status of KIR3DL1/KIR3DLS1 (data not shown), nor was there evidence of an association of clinical outcome linked to KIR3DL1/KIR3DS1 status together with the HLA-Bw4 ligand isoforms (data not shown). Rather, the mere presence of KIR3DL1 together with its ligand, HLA-Bw4, seemed to influence patients’ response to immunotherapy in both clinical trials. These observations will require validation in a separate study.

In conclusion, this work sheds further light on the role of KIR receptors on NK cells in the antitumor response to immunotherapeutic mAbs. We demonstrate that the KIR3DL1/HLA-Bw4 axis influences response to tumor-targeted mAbs in two separate clinical trials and that the presence of the B-Bw4-T80 isoform or the A-Bw4 isoform is associated with improved response to mAb-based immunotherapy, while the presence of the B-Bw4-I80 isoform is not.

## Ethics Statement

This study was carried out in accordance with the recommendations of University of Wisconsin Health Sciences Institutional Review Board with written informed consent from all subjects. All subjects gave written informed consent in accordance with the Declaration of Helsinki. The protocol was approved by the University of Wisconsin Health Sciences Institutional Review Board.

## Author Contributions

Each author made substantial contributions to the conception and/or design of this research, including the acquisition, analysis, and/or interpretation of data for the work; the drafting of this manuscript, including critical revisions important intellectual content, were shared duties by all authors; each author submitted final approval of this manuscript as submitted to be published; each author is in agreement to be accountable for all aspects of the work in ensuring that questions related to the accuracy or integrity of any part of the work are appropriately investigated and resolved (AE, WW, PR, LC, KK, EM, YS, JH, WL, AN, FH, MH, JM, JP, MO, JM, AG, BK, AY, and PS).

## Conflict of Interest Statement

The authors declare that the research was conducted in the absence of any commercial or financial relationships that could be construed as a potential conflict of interest.
