# Supplementary material for: HLA-Bw4-I-80 Isoform Differentially Influences Clinical Outcome As Compared to HLA-Bw4-T-80 and HLA-A-Bw4 Isoforms in Rituximab or Dinutuximab-Based Cancer Immunotherapy
Source: Front Immunol. 2017 Jun 12;8:675. doi: 10.3389/fimmu.2017.00675 (PMC5466980; doi:10.3389/fimmu.2017.00675)
Supplement: Supplementary file 1 [file Table_1.DOCX]

**Supplemental Table 1. KIR3DL1 and HLA-Bw4 isoform genotypes that compose KIR3DL1/HLA-Bw4-Isoforom Genotypes.**
