# Supplementary material for: HLA-Bw4-I-80 Isoform Differentially Influences Clinical Outcome As Compared to HLA-Bw4-T-80 and HLA-A-Bw4 Isoforms in Rituximab or Dinutuximab-Based Cancer Immunotherapy
Source: Front Immunol. 2017 Jun 12;8:675. doi: 10.3389/fimmu.2017.00675 (PMC5466980; doi:10.3389/fimmu.2017.00675)
Supplement: Supplementary file 2 [file Table_2.DOCX]

**Supplemental Table 2. Absence of Linkage Disequilibrium Between HLA-A-Bw4 and the HLA-Bw4-T80 and HLA-Bw4-I80 Isoforms.** The number of patients positive or negative for each of the 3 HLA-Bw4 isoforms from each of the 2 clinical studies are shown. As the HLA-A-Bw4 isoform is located at a separate, linked, locus to the location of the HLA-Bw4-T80 and HLA-Bw4-I80 Isoforms, we evaluated whether the presence or absence of the HLA-A-Bw4 isoform was significantly associated with the presence or absence of either the HLA-Bw4-T80 isoform or the HLA-Bw4-I80 Isoform. There was no such significant association, and thus no significant evidence of linkage disequilibrium. Chi-square tests were used to test for linkage disequilibrium.
